# Design of morphing patterns in knitted SMA textile actuators via knitting codes

**DOI:** 10.1038/s41598-026-50219-5

**Published:** 2026-04-27

**Authors:** Ju-Hee Lee, Yeji Han, Eunsol Park, Min-Woo Han

**Affiliations:** 1 Department of Mechanical Engineering, Dongguk University, Seoul, 04620 South Korea; 2 Department of Mechanical, Robotics and Energy Engineering, Dongguk University, Seoul, 04620 South Korea

**Keywords:** Engineering, Materials science

## Abstract

Shape memory alloys (SMAs) are widely used as soft actuators due to their shape memory effect, variable stiffness, and simple actuation via Joule heating. To achieve larger and more diverse deformations, SMA wires can be integrated as fibers into textile structures, enabling the fabrication of textile actuators using conventional textile manufacturing methods. These actuators provide high compliance and ergonomic conformability, making them more human-friendly than other types of soft actuators. Nevertheless, few studies have examined their modal behavior, limiting the application of SMA-based textile actuators. This study focuses on knitted SMA-based textile actuators and investigates nine morphing patterns—six plane-based and three band-based—designed with knitting codes. The actuators exhibited deformations that matched the predicted shapes. In addition, the plane-based patterns were analyzed to examine the relationship between loop transition boundaries and deformation axes in controlling bending angles. Demonstrations of knitted morphing flowers highlighted the scalability of the designs. Furthermore, quantitative evaluation of actuation forces in horizontal and vertical directions confirmed that actuator performance can be tuned through structural design.

**Supplementary Information:**

The online version contains supplementary material available at 10.1038/s41598-026-50219-5.

## Introduction

Humans spend most of their lives interacting with textile structures such as clothing, bedding, and shoes. Textiles are closely connected with human history, and over thousands of years of technical breakthroughs, they have evolved from serving basic purposes such as skin protection and heat conservation to fulfilling more advanced needs, such as expressing individuality and enhancing sports performance^1,2^. Recently, with a focus on the fact that textiles, in the form of clothing, are always in contact with human body, there has been growing interest in enhancing their functionality for various purposes, such as fashion, healthcare, rehabilitation, and gaming^3–5^. In other words, future textile technology would aim to create interactive textiles that can function as wearable actuators or sensors. To achieve this, there have been developments of various types of textile sensors at a commercial level, such as heartbeat monitoring sensors^6,7^, temperature sensors^8,9^, humidity sensors^10–12^. However, the development of textile actuators is relatively challenging. The materials used for the actuators should be compliant enough for wearability^13,14^ However, bulk soft materials, such as elastomers, hydrogels, and soft polymer foams, generally exhibit low energy density and specific power when implemented in compact or thin configurations, which limits their effectiveness as actuators for wearable devices^15^. To address this limitation, numerous studies have explored compliant textile actuators, either by integrating smart materials into textile structures or by using textiles themselves as functional actuator components.

Various types of smart materials have been used to develop compliant textile actuators. Pneumatic or hydraulic artificial muscles are a type of soft actuator that generates movement by expanding and contracting chambers with compressed air or liquid. Its strength and flexibility are comparable to human muscles^16–18^. Belforte et al.^19^ introduced linear upper limb movements with pneumatic fiber muscles, and Phan et al.^20^ achieved 18% strain with woven hydraulic artificial muscles. Liquid crystal elastomers (LCEs) are flexible materials that respond to external stimuli such as temperature, light, and electric fields^21,22^. Sun et al.^23^ developed textile LCE actuators that can twist and fold using knitting patterns, and Silva et al.^24^ proposed active textile fabrics by weaving LCEs that exhibit a blocking force of 1–2 N.

Shape memory alloys (SMAs) are a type of smart material that recover their trained configuration at high temperatures^25^. Due to their advantageous characteristics, SMAs are used in various applications from daily products such as shape memory eyeglass frames to wearable devices, surgical equipment, and aerospace components^26–28^. Park et al.^29^ developed a lightweight fabric muscle by bundling SMA springs. The muscle achieved an actuating force of over 100 N with a 50% contraction strain at 70 °C. Shin et al.^30^ proposed a knitted textile soft gripper using a knitting technique. By designing the gripper based on the investigation, the knitted textile gripper successfully lifted objects of various shapes and weights.

While these smart materials have contributed to the advancement of textile actuators, each presents distinct limitations, including external equipment dependence, performance trade-offs, and restricted design versatility. Pneumatic or hydraulic artificial muscles require external pump and valve systems, making the overall system heavy, limiting wearability and convenience. LCEs have a trade-off between actuation stress and actuation strain, both of which are key factors in determining the performance of the actuator, thereby limiting their application to wearable actuators^31^. On the other hand, SMAs exhibit high work density, which allows for compact and efficient actuator designs suitable for textile applications^25^. SMAs have high durability, repeatability, and reliability^32,33^, and they can be manufactured in the form of thin wires that can serve as fibers—the building blocks of textile structures—which can be used to produce textile structures through conventional textile manufacturing methods. Due to these advantageous characteristics, SMAs can be expected to play a key role in shaping the future of textile actuators.

Recently, multiple studies on textile actuators have been conducted using SMA wires as fibers to create textile structures. Han et al.^34^ developed woven-type SMA textile actuators achieving three-dimensional surface morphing and demonstrated the feasibility of machine-based manufacturing using commercial weaving machines. A related study^35^ used knitting techniques to produce four knitting patterns and designed soft textile actuators that mimic blooming knit flowers. Lee and Han^36^ developed chain structure based textile actuators by interlocking loop structures using SMA fibers, demonstrating torsional deformation behaviors and soft gripper applications. In particular, knitting demonstrates unique capabilities due to its loop architecture, characterized by its inherent void space, which allows for full utilization of this spatial volume during deformation, enabling high strain capabilities. These strengths make knitted textile actuators highly promising as interactive textiles, with potential applications in the fashion industry for individual expression, as well as in wearable rehabilitation devices that provide high wearability and customized morphing patterns.^37^ However, research on knitted SMA actuators has been largely confined to a limited set of morphing patterns, and the design parameters governing deformation behavior have not been systematically characterized. In particular, the influence of loop transition boundary orientation on bending angle remains unquantified, and actuation force distribution across multiple loading directions has not been evaluated.

To overcome these limitations, this study proposes nine distinct morphing patterns by varying knitting code arrangements. The target deformations were referenced from clothing features such as collars and skirt pleats, resulting in six plane-based patterns and three band-based patterns. The fabricated actuators were actuated at a constant current of 0.25 A, and the resulting deformation and bending angles were recorded over time. The relationship between a loop’s bending axis and its transition boundary is examined to understand its influence on bending behavior, providing quantitative insights into curvature control. In addition, actuation forces are evaluated in both vertical and horizontal directions to explore how curvature distribution affects force generation and directional response. Finally, the investigated morphing modes are demonstrated through morphing flower models, illustrating coordinated deformation enabled by multiple actuator units.

## Design and fabrication of knitted textile actuators

**Fig. 1:**
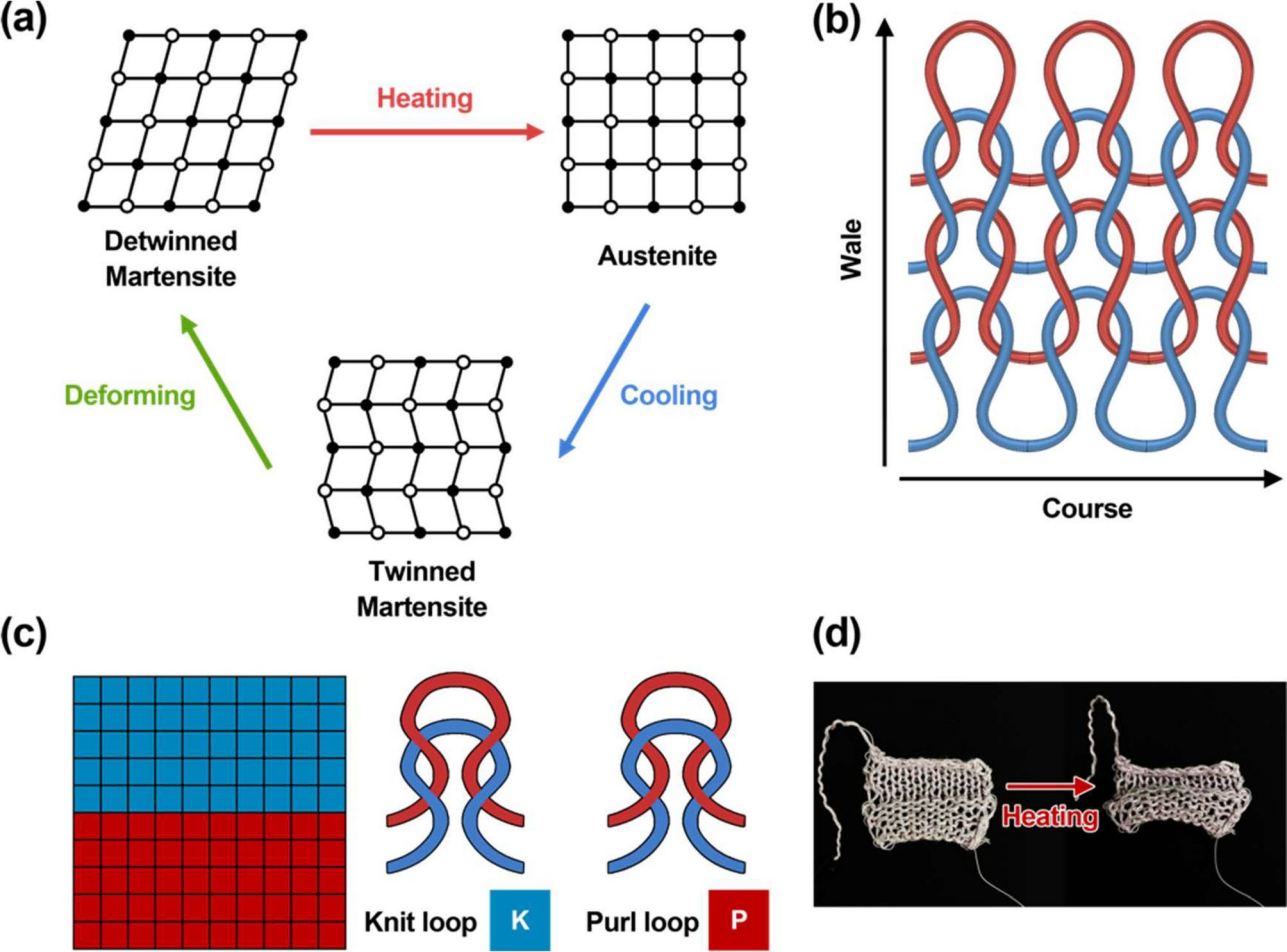
(a) Driving mechanism of SMAs, showing phase transitions from martensite to austenite due to temperature change. (b) Illustration of the knitting structure and the direction of courses (horizontal) and wales (vertical). (c) Illustration of a knitting code and the two fundamental loop types used in the knitting technique. The knitting code is a grid composed of knit (K) and purl (P) loops, indicated by blue and red cells, respectively. (d) An actuation example of a knitted SMA wire actuator fabricated according to knitting code shown in (c), demonstrating bending deformation upon heating.

The SMAs, a thermally responsive smart material, were used as the driving source for the knitted textile actuators. At room temperature, SMAs are in the martensite phase. When they absorb thermal energy and reach the austenite start temperature (*A*_s_), they transform into the austenite phase, triggering the shape memory effect and returning to their trained shape. SMAs in the austenite phase have unique mechanical behaviors such as high stiffness and superelasticity. Conversely, when the heat supply is cut off and the SMAs are cooled down to reach the martensite final temperature (*M*_f_), the SMAs undergo a phase change to martensite, resulting in lower stiffness and becoming easily deformable under relatively small forces. Figure 1(a) illustrates the working mechanism of the SMAs, showing phase transitions from martensite to austenite with temperature changes. Table 1 presents the mechanical properties of the SMA used for the knitted textile actuators^38^. In this study, Ni-Ti SMA wire (55 wt% Ni and 45 wt% Ti) with a core diameter of 200 *µ*m was used (Flexinol^®^, Dynalloy Inc.). The 200 μm diameter was selected to balance electrical resistance, actuation force, and thermal response as shown in Supplementary Table S1.

**Table 1:** Mechanical properties of SMA wires used for the actuators^38^.

Properties	Value	Unit
Martensite modulus	26.3	GPa
Austenite modulus	75	GPa
Thermal coefficient	0.55	GPa
Martensite start temperature (*M*_*s*_)	52	◦ C
Martensite finish temperature (*M*_*f*_ )	42	◦ C
Austenite start temperature (*A*_*s*_)	68	◦ C
Austenite finish temperature (*A*_*f*_ )	78	◦ C

SMAs can be manufactured in the form of wires and used as fibers—the fundamental building blocks of knitting—enabling the fabrication of patches using textile manufacturing techniques. Since knitting techniques use pre-defined knitting codes for consistent production, machine-based fabrication is feasible with commercial knitting machines, as demonstrated in Supplementary Figure S1. The knitted structure, as shown in Fig. 1(b), is composed of loops. Vertically arranged loops are referred to as wales, while horizontally arranged loops are referred to as courses, which are used to count the number of loops in each direction. In knitting, there are two types of loops: knit loop (K) and purl loop (P). A knit loop has the head of the upper loop positioned above the head of the lower loop, whereas a purl loop has the head of the upper loop beneath the head of the lower loop. A knitting code is a design blueprint in knitting that describes which loop has to be included at specific position. Figure 1(c) shows an example of a knitting code along with simple illustrations of purl and knit loops.

The knitted textile actuators used in this study are fabricated to a structure size of 10 courses × 10 wales, where active and inactive fibers are knitted together according to knitting codes. When textile actuators are constructed solely from SMA wires, the smooth wire surface leads to inter-wire slippage, causing the knitted structure to become unstable and prone to unraveling. In contrast, the use of polyester-wrapped SMA wires (active wires) increases interfacial friction, thereby enhancing the structural robustness of the knitted actuator. This improvement, however, introduces a trade-off, as the polyester insulation layer impedes heat dissipation and reduces the cooling rate, as evidenced in Supplementary Figure S2. The polyester wrapping has a thickness of approximately 100 μm, resulting in a total wire diameter of 400 μm. The inactive fiber is a knitting yarn composed of 85% cotton and 15% polyester, which deforms passively under external stimuli while contributing additional friction to further stabilize the structure. In addition, the polyester wrapping provides electrical insulation between adjacent wires, preventing localized overheating even when wires come into contact during fabric deformation, as confirmed by thermal imaging (Supplementary Figure S3). Figure 1(d) illustrates the pre- and post- actuation states of the knitted textile actuator fabricated according to the knitting code in Fig. 1(c). The current for actuation was set to 0.25 A, selected as an intermediate condition that enables stable and gradual actuation while allowing detailed observation of the morphing process (Supplementary Figure S3). The electrical resistance and Joule heating energy of each actuator are summarized in Supplementary Tables S2 and S3.

**Fig. 2:**
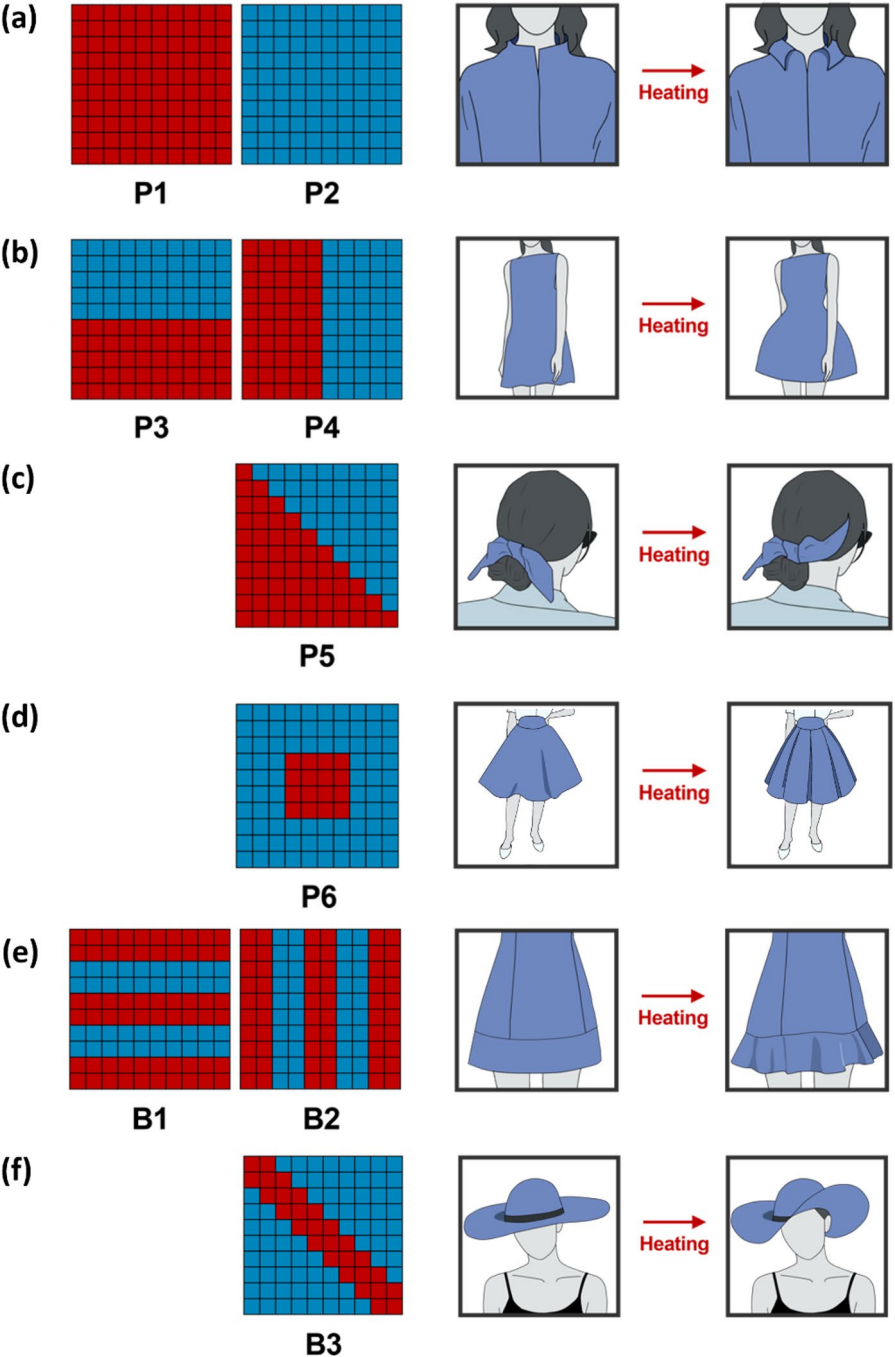
Knitting code patterns and corresponding actuated shapes for interactive garment applications. Plane-based patterns: (a) P1 and P2 showing upward/downward curling, (b) P3 and P4 demonstrating alternating bends, (c) P5 with diagonal deformation, (d) P6 with flat central region and curved edges. Band-based patterns: (e) B1 and B2 showing alternating bands with pleats, (f) B3 with diagonal band for convex shape.

Han et al.^30^ reported that knitted textile actuators composed exclusively of knit loops produce upward curling, while those composed exclusively of purl loops produce downward curling. Based on the fundamental deformation characteristics of single-loop patterns, knitting codes for nine target deformations were proposed, and the corresponding actuators were fabricated. The target deformations are inspired by the design of clothing in the fashion industry, such as jacket collars, skirt pleats, and the waistline of dresses.The nine patterns are divided into six plane-based patterns and three band-based patterns. As shown in Fig. 2(a-d), plane-based patterns (P1-P6) refer to patterns where purl loops are arranged in a planar form.

On the other hand, band-based patterns (B1-B3), as shown in Fig. 2(e, f), refer to patterns where purl loops are included in a band form. The nine knitted textile actuators were then fabricated according to their knitting codes and their morphing modes were experimentally investigated. The investigation of morphing modes involves the following: (1) recording the deformation of the actuators over time and verifying whether the target deformation was achieved, and (2) investigating the effect of the relationship between the loop transition boundary line and the deformation axis of a single loop on the bending angle of the actuator, (3) quantitatively measuring the actuation forces to assess actuation performance across patterns, and (4) demonstrating their application as knitted morphing flowers to validate practical versatility.

## Investigation on morphing modes with knitting codes

**Fig. 3:**
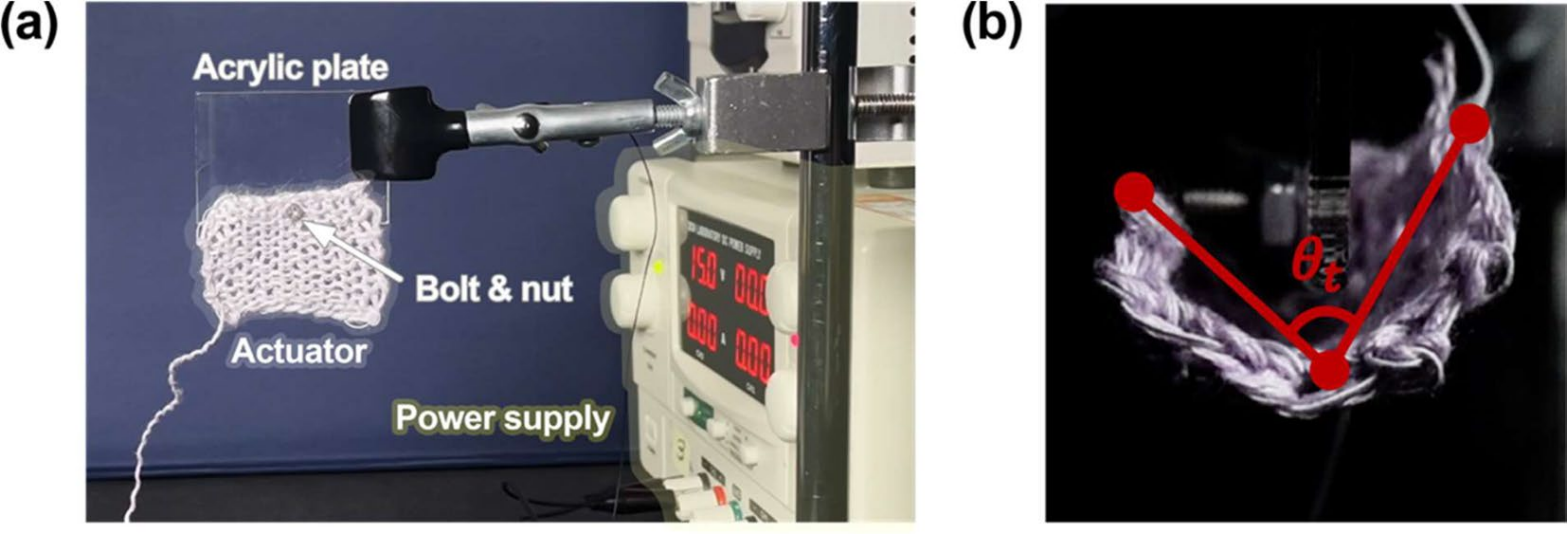
(a) Experimental setup. (b) Measurement of the angle *θ*_*t*_.

To investigate the morphing mode of the nine knitting codes, the actuation was recorded with cameras. Figure 3(a) shows the experimental setup. The acrylic plate was first clamped to the support stand, after which the actuator was mounted to the plate through a punched hole using bolts and nuts. To prevent interference with the actuator’s deformation, only the topmost portion of the actuator was fixed to the lower side of the acrylic plate, leaving the remainder of the actuator unconstrained during actuation. Then the electrodes were connected to the power supply, and the operation of the actuator was recorded from the front and side views.

All actuators were operated under the same current of 0.25 A, and the maximum deformation was considered to be achieved when the actuator’s shape was maintained in the same configuration for more than 3 s. The deformation of the actuators over time was investigated for six plane-based actuators and three band-based actuators. Additionally, for plane-based actuators except for P5, the bending angle was recorded to investigate the effect of the relationship between the loop transition boundary line and the deformation axis of a single loop. Figure 3(b) shows the measurement of angle *θ*_*t*_, which is defined as the angle connecting one end of the actuator, the central point of the actuator, and the other end of the actuator at a specific time *t*. To record the bending angle change at a specific time *t* (*θ*_*t, b*_) relative to the angle at *t* = 0 (*θ*_0_), we acquired *θ*_*t, b*_ = *θ*_0_ *− θ*_*t*_ as a function of time.

### Plane-based actuators

Plane-based actuators are based on knitting codes that include purl loops in a planar configuration. These include P1 and P2, which are composed of one loop type – Purl or knit loop; P3 and P4, where the loop transition boundary is parallel to the edge of the patch; P5, where the loop transition boundary forms a 45-degree angle with the edge of the patch; and P6, which includes purl loops in the form of an island. Supplementary Video S1 shows the deformation of plane-based actuators.

**Fig. 4:**
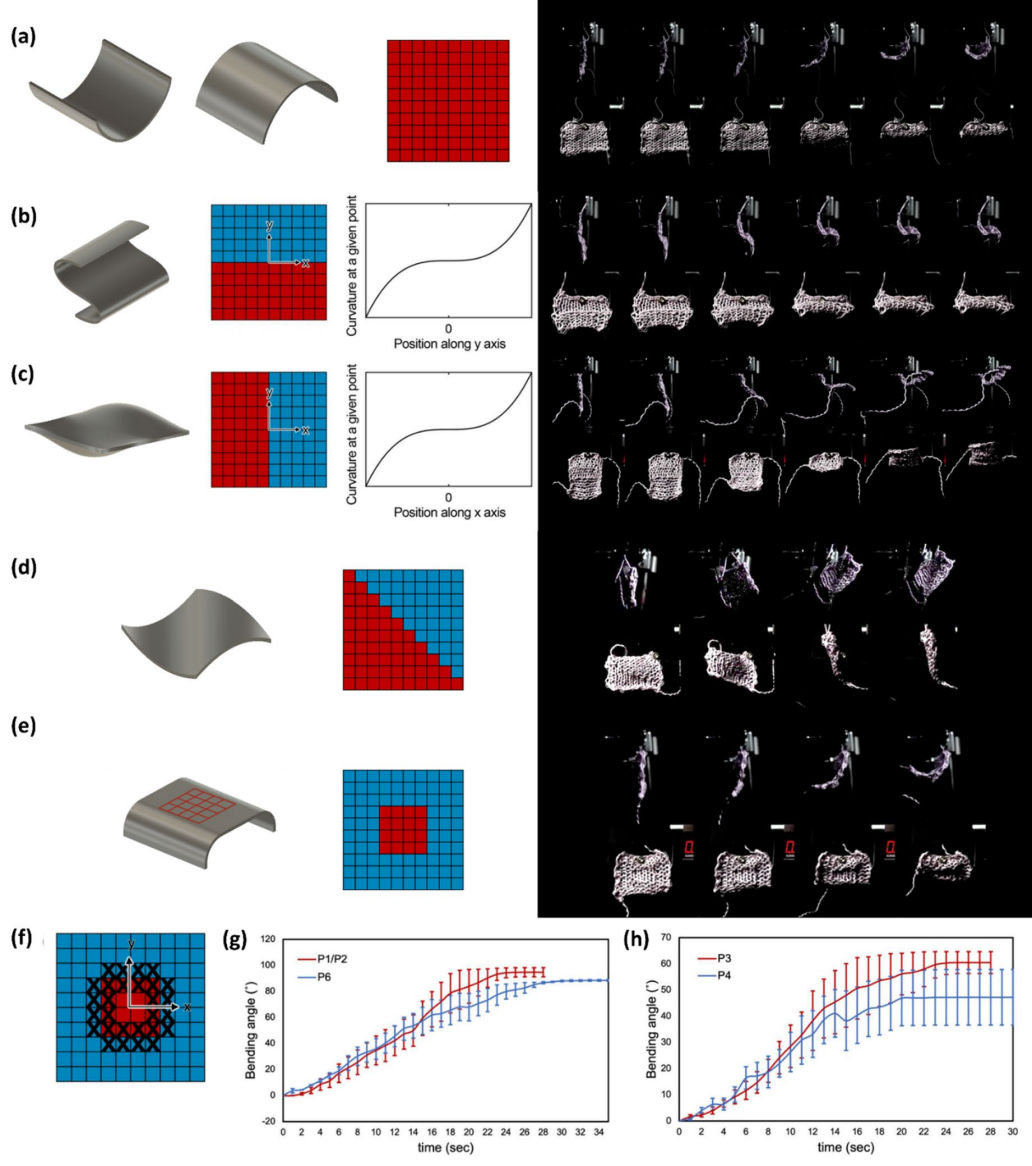
Deformation of plane-based SMA knitted actuators. (a–e) From left to right: expected deformation geometry, knitting pattern with loop transition boundaries, and temporal evolution (side and front views; scale bars = 10 mm). Actuation sequences were recorded at 5 s intervals (a–c) and 10 s intervals (d–e). (a) P1/P2. (b) P3. (c) P4. (d) P5. (e) P6. (f) Loop interaction diagram at transition boundaries (X marks indicate deformation cancellation). (g, h) Bending angle versus time: (g) P1/P2 and P6; (h) P3 and P4. Error bars represent standard deviation (*n* = 3).

P1 and P2 were designed with reference to a shirt collar transitioning from a flat to a curved shape. They are composed solely of purl or knit loops, and since purl and knit loops have the same configuration when flipped, P1 and P2 are essentially the same actuators. P1 deforms into a convex shape downward and P2 into a convex shape upward. The expected deformations are as illustrated in Fig. 4(a). To investigate the morphing mode, P1 was fabricated according to the knitting code and actuated.

Figure 4(a) shows the deformation of P1 over time. Over a period of 28 s, the actuator gradually deformed into a downward-convex shape, consistent with the expected behavior and demonstrating agreement in both curvature direction and overall deformation profile. Since P2 is the flipped version of P1, the experimentally observed deformation of P2 likewise agrees with the predicted upward-convex deformation. By referring to P1 and P2 as fundamental patterns, other patterns were designed. These results confirm that the deformation behavior of P1 and P2 follows the design principles defined by the knitting code arrangement, validating their role as fundamental patterns for constructing more complex morphing modes.

P3 and P4 were designed with reference to the shape of a dress transitioning from a straight waistline to a cinched waistline. The difference between P3 and P4 lies in whether the loop transition boundary is parallel to (P3) or perpendicular to (P4) the bending axis of the single loop. In the case of P3, while the upper and lower loops bend in opposite directions at the loop transition boundary, the adjacent loops on the same wale line deform in the same direction. Therefore, the upper and lower loops at the boundary are likely to deform independently without interfering with each other. Therefore, P3 was expected to have a bending deformation with a high-curvature S-shape, as shown in Fig. 4(b). This behavior can be understood from the deformation of individual loops, which primarily bend out of plane about a horizontal axis (i.e., along the course direction). In P3, where the loop transition boundary is parallel to this bending axis, adjacent loops belonging to different regions are arranged along the vertical (wale) direction. As a result, their bending occurs about spatially separated axes, allowing each loop to deform without directly constraining its neighbors and enabling the development of larger curvature.

In contrast, in the case of P4 the loops at the loop transition boundary bend in opposite directions and interfere with each other’s deformation. Specifically, the loop transition boundary is perpendicular to the bending axis, and adjacent loops are arranged along the horizontal (course) direction. In this configuration, neighboring loops attempt to bend in opposite directions about the same axis. Because the loops are mechanically connected, this creates geometric incompatibility, where one loop tends to bend upward while the adjacent loop tends to bend downward. This mismatch leads to mutual constraint and partial suppression of deformation, resulting in reduced overall curvature. Accordingly, P4, as shown in Fig. 4(c), was expected to exhibit a bending deformation with an S-shape that has near-zero curvature at the boundary and higher curvature further away from the boundary.

Figure 4(b) shows the deformation of P3 over time in the experiment. It took 28 s for the deformation to reach the final configuration, during which P3 exhibited an S-shaped bending consistent with the expected deformation. P4 deformed to match the expected shape over 30 s as shown in Fig. 4(c). This slightly longer deformation time of P4 may be associated with the orientation of the loop transition boundary, where the perpendicular boundary line introduces greater interaction between adjacent loops, slowing the deformation process. The bending angles were recorded for the upper C-shaped part of P3 and P4. As shown in Fig. 4(h), P3 had a greater maximum bending angle than P4. This indicates that when the loop transition boundary line is parallel to the deformation axis of the single loop, the deformations of adjacent purl and knit loops are almost independent. On the other hand, when they are perpendicular, the deformations of the adjacent knit and purl loops interfere with each other and result in a smaller bending angle for the actuator. These results confirm that the deformation behavior of P3 and P4 follows the design principles defined in Fig. 2, demonstrating that boundary orientation governs both the qualitative deformation mode and the resulting bending magnitude.

P6 is an actuator designed with reference to a box-pleated skirt, which transforms from a flat shape into a uniform pattern of vertical pleats. It includes a 4 × 4 grid of purl loops in the center. Since the loops on the upper and lower loop transition boundaries are expected to have independent deformations, there is no interference between the adjacent purl and knit loops at these boundaries. However, on the left and right loop transition boundaries, similar to P4, the movements of the purl and knit loops on either side cancel each other out, resulting in a near-zero slope, as illustrated in Fig. 4(e). P6 reached the steady state after 35 s, which was the longest among the plane-based actuators. This slower response is likely due to the constrained central region and opposing loop interactions, which restrict deformation and reduce the overall actuation rate. In the experiment, the knit loops above and below the 4 × 4 grid of purl loops deformed as expected, showing a bending deformation along the y-direction at the upper and lower sections. The loops that cannot deform independently are marked with X in Fig. 4(f), leaving only four purl loops that can bend downwards. The central region containing the 4 × 4 grid of purl loops remained flat with near-zero curvature, which aligns with the expected box pleated structure where the center remains flat while vertical pleats form at the boundaries. Therefore, P6 showed a bending deformation similar to P1 but with a flat central region, resulting in lower overall curvature. Figure 4(g) shows that the maximum bending angle of P1/P2 is greater than that of P6. This design demonstrates how strategically placed opposing loops create patterns for controlled deformation, expanding the design methodology. The reduced curvature observed in P6 suggests that this pattern is well suited for designing morphing structures that require gradual and smooth deformation, consistent with the intended design.

### Band-based actuators

Band-based actuators are based on knitting codes that include purl loops in a band form. B1 and B2 are actuators with alternating bands, while B3 is an actuator with diagonal bands. Supplementary Video S1 shows the actuation of the band-based actuators.

**Fig. 5:**
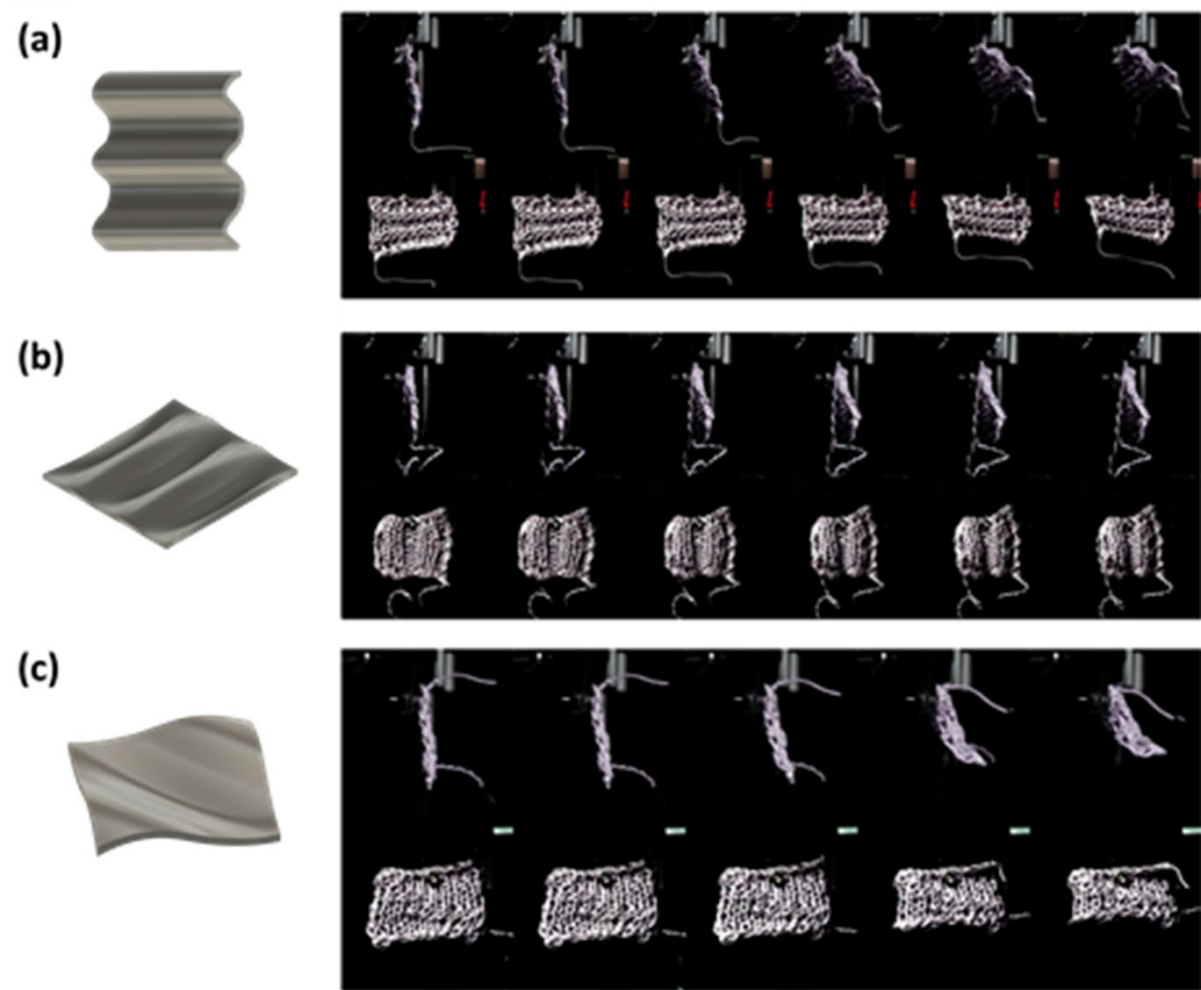
Expected deformation, knitting pattern, and temporal evolution (side/front views) for band-based actuators: (a) B1 at 5 s intervals. (b) B2 at 10 s intervals. (c) B3 at 10 s intervals.

B1 and B2 were designed with reference to a skirt transforming from a flat shape into a pleated shape. B1 consists of repeated P3 patterns, including alternating purl and knit loops in the wale direction. Since adjacent loops at the loop transition boundary undergo independent deformation, B1 was expected to achieve an alternating deformation pattern with upward and downward bending, similar to the deformation of P3. Figure 5(a) shows the deformation of B1 over time. The actuator deformed over 28 s, reaching the expected configuration.

B2 consists of repeated P4 patterns, including alternating purl and knit loops in the course direction. Figure 5(b) presents the expected deformation of B2. It was expected to have curvature approaching zero at the loop transition boundary. Additionally, since each band consists of two courses, achieving sufficient bending deformation can be difficult, based on the deformation observed in P6. The temporal evolution reveals that over 50 s, the actuator deformed as expected, contracting in the course direction.

B3 was designed with reference to a brim of a hat that changes from a flat shape into a convex shape in a diagonal direction. The band is composed of purl loops placed diagonally in the center. The deformation of the purl loops in the band was expected to be restricted by the neighboring knit loops, as they are not independent. However, unlike B2, where the loop transition boundary line is perpendicular to the deformation axis of the single loop, the boundary line in B3 forms a 45-degree angle. Therefore, B3 was expected to have a bending deformation greater than B2 but smaller than B1 as illustrated in Fig. 5(c). Over 45 s, the actuator deformed as anticipated, reaching the expected configuration.

In addition, a consistent trend was observed in the actuation time across different patterns. Patterns such as P1/P2, P3, and B1 exhibited similar deformation times, whereas P4 showed a slightly longer response, and P5 and B2 exhibited significantly longer actuation times. B3 showed an intermediate response between B1 and B2. This trend suggests that the orientation of the loop transition boundary influences the actuation dynamics. In particular, patterns with loop transition boundaries aligned along the vertical direction, such as P4 and B2, tend to exhibit longer deformation times. It is likely due to increased interaction and constraint between adjacent loops, which slows the propagation of deformation. These results indicate that, in addition to controlling deformation shape, the arrangement of loop transition boundaries can also be used to tune the actuation speed of knitted textile actuators.

## Correlation between curvature design and actuation force

**Fig. 6:**
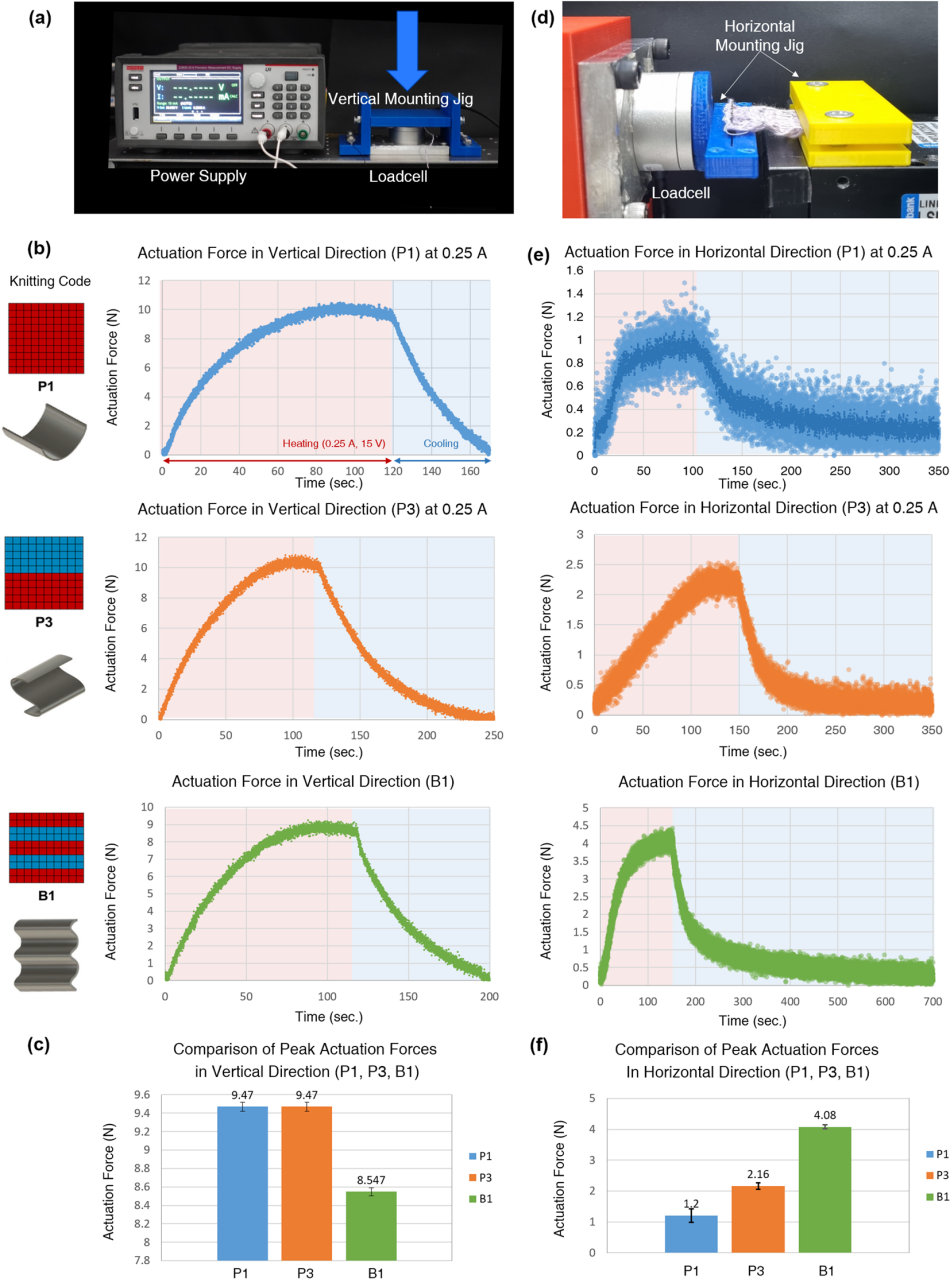
(a-c) Vertical direction: (a) Experimental setup showing the vertical mounting jig with load cell, (b) Actuation force measurements of P1, P3, and B1, (c) Comparison of peak actuation forces of P1, P3, and B1.(d-f) Horizontal direction: (d) Experimental setup showing the horizontal mounting jig with load cell, (e) Actuation force measurements of P1, P3, and B1, (f) Comparison of peak actuation forces of P1, P3, and B1.

To further investigate the actuation of the proposed knitting codes, we measured the horizontal and vertical forces generated by three representative patterns, P1, P3, and B1, each presenting a distinct curvature distribution. The patterns differed in the number of bending segments but were confined to the same overall area, allowing systematic comparison of curvature arrangement and force generation. The experimental setup includes the vertical and horizontal mounting jigs with textile patterns and a load cell, as shown in Fig. 6(a, d). P1 consists of a uniform loop structure that produces a single bending deformation. P3 includes a loop transition boundary that creates two curvatures in opposite directions. B1 is composed of five repetitive bending segments, resulting in the highest curvature density among the three. In addition, to evaluate the maximum actuation force of each pattern, experiments were conducted with thermal control, where the actuators were heated at 0.25 A until they reached their maximum force, followed by a cooling process until the force fully decreased to 0 N, enabling a comparison of the maximum actuation forces. The raw time–force datasets underlying the measurements shown in Fig. 6(b, e) are provided in Supplementary Data S1.

According to the actuation force measurements in the vertical direction, shown in Fig. 6(b, c), the peak force values were similar across all three patterns: P1 and P3 both generated 9.47 N, while B1 produced 8.55 N. These values represent the average of 10 repeated measurements, with an error range of approximately 0.25 N to 0.7 N. The variation in actuation force between patterns was minimal, indicating that the number of bends has a relatively minor influence on vertical force output.

In contrast, the horizontal actuation force, presented in Fig. 6(e, f), increased significantly with the number of bending segments. P1 had a peak force of 1.2 N, while P3 and B1 had 2.16 N and 4.08 N, respectively. This increasing trend is attributed to the accumulation of local loop contractions, which enhance the overall horizontal shrinkage, representing an increase of up to 240% (from 1.2 N to 4.08 N). Directional force modulation based on structural design can provide a useful strategy for developing wearable systems and soft actuators requiring programmable and anisotropic responses.

## Applications

As demonstration of this study, we developed morphing flower models, Pansy and Lily, to validate potential applications of knitted textile actuators beyond basic knit and purl patterns. These models have multiple petal actuators combined to form a single flower structure. With adjustments to the circuit architecture, each petal can be controlled independently or driven in coordination with other petals. Since multiple actuators can be easily combined in various ways as textile structures, knitted textile actuators are adaptable to a wide range of functions and applications.

**Fig. 7:**
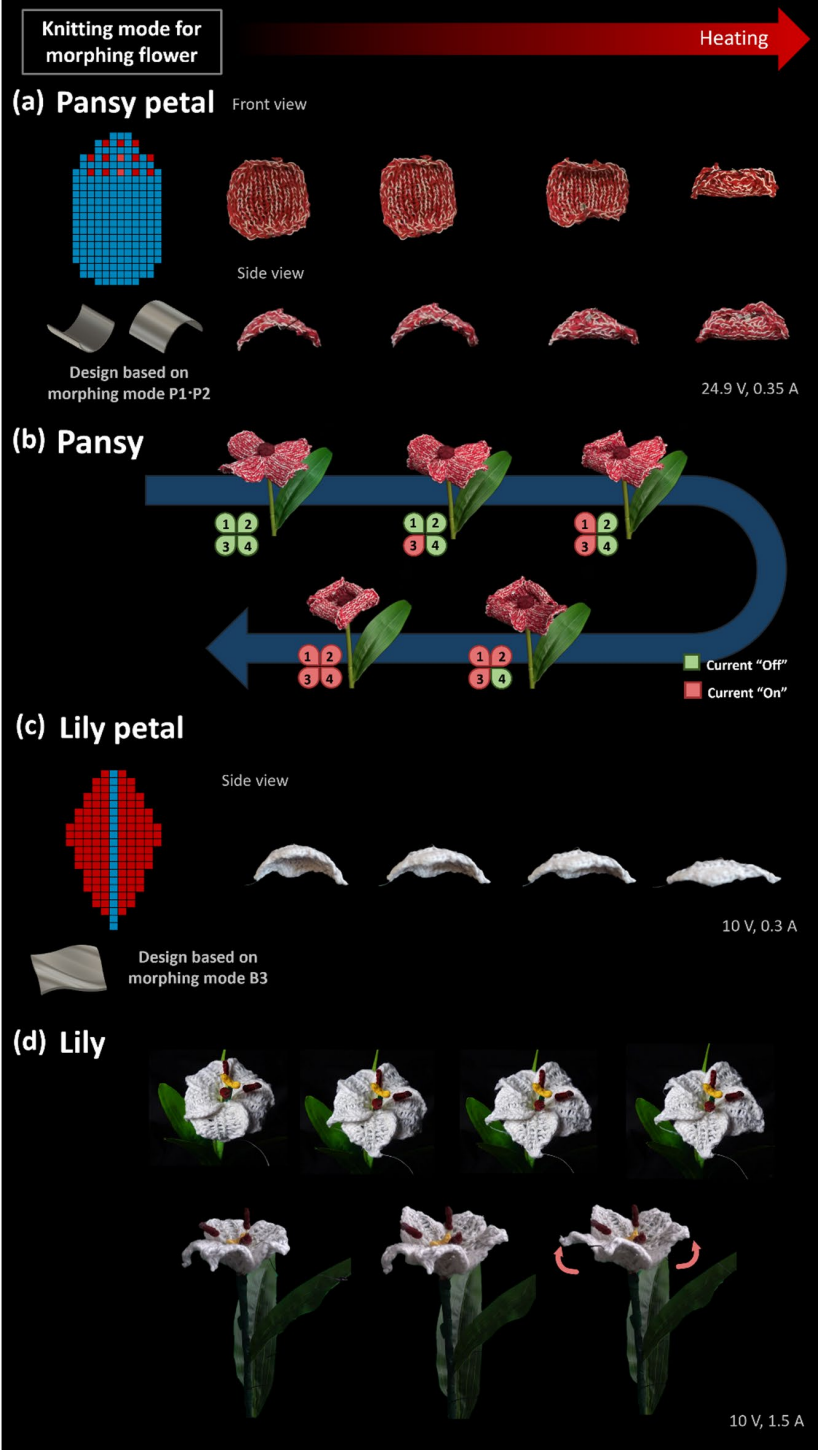
Morphing flower demonstrations using knitted SMA actuators. (a) Pansy model incorporating P1 and P2 patterns showing petal curling. (b) Sequential actuation of individual Pansy petals through combinatorial switching of four independent control channels. (c) Lily petal incorporating B3 pattern showing petal curling. (d) Lily incorporating B3 pattern showing diagonal deformation.

The Pansy and Lily models were designed based on the morphing modes of P1, P2, and B3 patterns. As shown in Fig. 7, these two models show deformation modes that align with the knitting code study. Pansy petals are designed based on the plane-based patterns, P1 and P2, having rounded shapes by gradually decreasing the number of courses in both the upper and lower parts of the petals. In addition, the Lily model was designed to have the alternating curvature of the band-based pattern B3. Lily petals feature rounded shapes formed by gradually decreasing the number of courses in both parts. A single loop at the uppermost part creates a pointed petal tip.

The actuation of each flower model can be controlled through current channel architecture. Current channels can be simply configured by connecting and separating SMA wires. This circuit arrangement enables two actuation modes: individual actuation through separated petal channels and simultaneous actuation through parallel connections. The actuation sequence can be programmed to achieve desired petal movements. Figure 7(b) shows the sequential actuation of the Pansy model through multiple petal channels.

To actuate all four petals simultaneously with a single signal, they were connected in parallel and driven with a current input of 1.4 A, as illustrated in the circuit schematic (Supplementary Figure S4), where each petal is configured as an individual branch. The electrical characteristics of a single petal, including power and energy consumption, are summarized in Supplementary Table S3. Instead of the square shape used for the P1 and P2 samples, the Pansy petals were modified to have rounded corners by gradually decreasing the number of courses in both the upper and lower parts. The curved deformation corresponds to the expected morphing modes in P1 and P2. The petal structure deformed from a flat surface to a curved shape, achieving the intended flower-closing motion as shown in Fig. 7(a). Additionally, when current was applied sequentially to the separate channels, the petals bent in sequence, demonstrating individual channel control as shown in Fig. 7(b).

The Lily petal design based on B3 pattern is shown in Fig. 7(c), featuring a diamond shape with a central single loop transition line. Figure 7(d) shows the full Lily model with five petals connected in parallel. Initial bending deformation was applied prior to electrical activation to enhance the visual folding effect of the central loop transition line. The actuator exhibited clear contraction along the course direction, where the central line folded, while the pre-existing bending straightened in the wale direction. Supplementary Video S1 illustrates these deformation characteristics during the actuation process.

Demonstrations of the Pansy and Lily models illustrate the potential of knitted textile actuators for diverse morphing modes and control. This study shows that complex deformation patterns—curvature, contraction, and directional deformation—can be achieved through separated channels. Additionally, customized movements can be designed by combining basic patterns through unique knitting codes, providing a platform for applying knitted textile actuators to various fields, including the fashion industry and wearable devices.

## Conclusion

In this study, nine morphing modes of knitted textile actuators were investigated by designing knitting codes and predicting their deformations. The fabricated actuators were then tested to observe the actual deformations. Additionally, the applications of morphing flowers were proposed. Since the actuators are fabricated based on predefined knitting codes, they have high repeatability. This approach can be extended to machine-based fabrication using commercial knitting machines. Nine morphing modes, referencing fashion-industry clothing shapes, exhibited distinct configurations with potential for future applications. The knitting codes were classified into six plane-based patterns and three band-based patterns based on the arrangement of purl loops in the knitting code. The actuators were fabricated according to the knitting codes and actuated at 0.25 A. The deformation was recorded over time during actuation, and the results matched the predicted shapes. Bending angles were analyzed based on the relationship between the loop transition boundary and the deformation axis of a single loop. The results showed that patterns with parallel alignment produced larger bending angles, while perpendicular alignment reduced bending due to interaction between adjacent loops. This can serve as a reference for adjusting the actuator’s curvature when designing knitted SMA actuators. Furthermore, actuation forces were quantitatively evaluated in the horizontal and vertical directions based on curvature distribution. The vertical force remained nearly constant across patterns (9.47 N for P1 and P3, and 8.55 N for B1), while the horizontal force increased with the number of bending segments, reaching up to 240% higher values. These results highlight the potential to modulate actuator performance through structural design modifications. Although this study presented and investigated various morphing modes of knitted textile actuators, further research is required to achieve a more complete understanding. While a physical model can allow prediction of deformations without experiments, the structural complexity and stress distribution of textiles make it difficult to establish an accurate constitutive model. To address this limitation, future work will employ finite element method (FEM) simulations incorporating material properties (e.g., stiffness, elasticity, strength) and electric fields, providing an advanced approach for state estimation and control.

## Supplementary Information

Below is the link to the electronic supplementary material.

Supplementary Material 1

Supplementary Material 2

Supplementary Material 3

## Data Availability

All data supporting the findings of this study are included in this published article and its Supplementary Information files. Additional raw datasets are available from the corresponding author on reasonable request.

## References

[1] Dhamija, J. *Textile: The Non-Verbal Language* (Telos Art Publishing, 2003).

[2] Snodgrass, M. E. *World Clothing and Fashion: An Encyclopedia of History, Culture, and Social Influence* (Routledge, 2015).

[3] Heo, J. S., Eom, J., Kim, Y. H. & Park, S. K. Recent progress of textile-based wearable electronics: a comprehensive review of materials, devices, and applications. *Small* **14**, 1703034 (2018). 10.1002/smll.201703034 29205836

[4] Komolafe, A. et al. E-textile technology review–from materials to application. *Ieee Access.* **9**, 97152–97179 (2021).

[5] Tyler, D. et al. Wearable electronic textiles. *Text. Prog.* **51**, 299–384 (2019).

[6] Yang, X. et al. Textile fiber optic microbend sensor used for heartbeat and respiration monitoring. *IEEE Sens. J.* **15**, 757–761 (2014).

[7] Quandt, B. M. et al. Body-monitoring with photonic textiles: a reflective heartbeat sensor based on polymer optical fibres. *J. R. Soc. Interface*. **14**, 20170060 (2017). 28275123 10.1098/rsif.2017.0060 PMC5378150

[8] Husain, M. D. & Kennon, R. Preliminary investigations into the development of textile based temperature sensor for healthcare applications. *Fibers* **1**, 2–10 (2013).

[9] Kuzubasoglu, B. A., Sayar, E. & Bahadir, S. K. Inkjet-printed cnt/pedot: Pss temperature sensor on a textile substrate for wearable intelligent systems. *IEEE Sens. J.* **21**, 13090–13097 (2021).

[10] Kinkeldei, T., Zysset, C., Cherenack, K. & Tröster, G. A textile integrated sensor system for monitoring humidity and temperature. In *2011 16th International Solid-State Sensors, Actuators and Microsystems Conference* 1156–1159 (IEEE, 2011).

[11] Ma, L. et al. Full-textile wireless flexible humidity sensor for human physiological monitoring. *Adv. Funct. Mater.* **29**, 1904549 (2019).

[12] Xu, L. et al. Coolmax/graphene-oxide functionalized textile humidity sensor with ultrafast response for human activities monitoring. *Chem. Eng. J.* **412**, 128639 (2021).

[13] O’Neill, C. T., McCann, C. M., Hohimer, C. J., Bertoldi, K. & Walsh, C. J. Unfolding textile-based pneumatic actuators for wearable applications. *Soft Rob.* **9**, 163–172 (2022). 10.1089/soro.2020.0064 33481682

[14] Wu, Y., Yang, Y., Li, C., Li, Y. & Chen, W. Flexible and electroactive textile actuator enabled by pedot: Pss/mof-derivative electrode ink. *Front. Bioeng. Biotechnol.* **8**, 212 (2020). 32266239 10.3389/fbioe.2020.00212 PMC7096353

[15] Perera, O., Liyanapathirana, R., Gargiulo, G. & Gunawardana, U. A review of soft robotic actuators and their applications in bioengineering, with an emphasis on hasel actuators’ future potential. *Actuators* **13**, 524 (2024).

[16] Walker, J. et al. Soft robotics: a review of recent developments of pneumatic soft actuators. In *Actuators, vol. 9* 3 (2020).

[17] El-Atab, N. et al. Soft actuators for soft robotic applications: a review. *Adv. Intell. Syst.* **2**, 2000128 (2020).

[18] Jamil, B., Lee, S. & Choi, Y. Conductive knit-covered pneumatic artificial muscle (k-pam) actuator. In *IEEE/RSJ International Conference on Intelligent Robots and Systems (IROS)* 1476–1481 (2018). 10.1109/IROS.2018.8594510.

[19] Belforte, G., Eula, G., Ivanov, A. & Sirolli, S. Soft pneumatic actuators for rehabilitation. *Actuators* **3**, 84–106 (2014).

[20] Phan, P. T. et al. Fabrication, nonlinear modeling, and control of woven hydraulic artificial muscles for wearable applications. *Sens. Actuators A: Phys.* **360**, 114555. 10.1016/j.sna.2023.114555 (2023).

[21] Kularatne, R. S., Kim, H., Boothby, J. M. & Ware, T. H. Liquid crystal elastomer actuators: synthesis, alignment, and applications. *J. Polym. Sci. Part. B: Polym. Phys.* **55**, 395–411 (2017).

[22] Jiang, H., Li, C. & Huang, X. Actuators based on liquid crystalline elastomer materials. *Nanoscale* **5**, 5225–5240 (2013). 23648966 10.1039/c3nr00037k PMC3697106

[23] Sun, J., Liao, W. & Yang, Z. Additive manufacturing of liquid crystal elastomer actuators based on knitting technology. *Adv. Mater.* **35**, 2302706 (2023). 10.1002/adma.202302706 37278691

[24] Silva, P. E. et al. Active textile fabrics from weaving liquid crystalline elastomer filaments. *Adv. Mater.* **35**, 2210689 (2023). 10.1002/adma.202210689 36639143

[25] Jani, J. M., Leary, M., Subic, A. & Gibson, M. A. A review of shape memory alloy research, applications and opportunities. *Mater. Des. (1980–2015)*. **56**, 1078–1113 (2014).

[26] Seelecke, S. Mu¨ ller, I. Shape memory alloy actuators in smart structures: modeling and simulation. *Appl. Mech. Rev.* **57**, 23–46 (2004).

[27] Özkul, I., Kurgun, M. A., Kalay, E., Canbay, C. A. & Aldas¸, K. Shape memory alloys phenomena: classification of the shape memory alloys production techniques and application fields. *Eur. Phys. J. plus*. **134**, 585 (2019).

[28] Srivastava, R., Alsamhi, S. H., Murray, N. & Devine, D. Shape memory alloy-based wearables: a review, and conceptual frameworks on hci and hri in industry 4.0. *Sensors* **22**, 6802 (2022). 36146151 10.3390/s22186802 PMC9504003

[29] Park, S. J., Kim, U. & Park, C. H. A novel fabric muscle based on shape memory alloy springs. *Soft Rob.* **7**, 321–331 (2020). 10.1089/soro.2018.0107 31724903

[30] Shin, J., Han, Y. J., Lee, J. H. & Han, M. W. Shape memory alloys in textile platform: smart textile-composite actuator and its application to soft grippers. *Sensors* **23**, 1518 (2023). 36772558 10.3390/s23031518 PMC9919340

[31] Sun, D. et al. Toward application of liquid crystalline elastomer for smart robotics: state of the art and challenges. *Polymers* **13**, 1889 (2021). 34204168 10.3390/polym13111889 PMC8201031

[32] Costanza, G. & Tata, M. E. Shape memory alloys for aerospace, recent developments, and new applications: a short review. *Materials* **13**, 1856 (2020). 32326510 10.3390/ma13081856 PMC7216214

[33] Sharma, N., Raj, T. & Jangra, K. Applications of nickel-titanium alloy. *J. Eng. Technol.* **5**, 1 (2015).

[34] Han, M. W., Kim, M. S. & Ahn, S. H. Shape memory textile composites with multi-mode actuations for soft morphing skins. *Compos. Part. B: Eng.* **198**, 108170 (2020).

[35] Han, M. W. & Ahn, S. H. Blooming knit flowers: loop-linked soft morphing structures for soft robotics. *Adv. Mater. (Deerfield Beach Fla)* **2017**, 29 (2017). 10.1002/adma.201606580 28165168

[36] Lee, J. H. & Han, M. W. Design and evaluation of smart textile actuator with chain structure. *Materials* **16**, 5517 (2023). 37629808 10.3390/ma16165517 PMC10456553

[37] Seyedin, S., Moradi, S., Singh, C. & Razal, J. M. Continuous production of stretchable conductive multifilaments in kilometer scale enables facile knitting of wearable strain sensing textiles. *Appl. Mater. today*. **11**, 255–263 (2018). 10.1016/j.dib.2018.04.090 PMC5998204 29904677

[38] Dynalloy, I. Flexinol® actuator wire technical and design data (2026).

