# Supplementary material for: Design of morphing patterns in knitted SMA textile actuators via knitting codes
Source: Sci Rep. 2026 Apr 27;16:19462. doi: 10.1038/s41598-026-50219-5 (PMC13287490; doi:10.1038/s41598-026-50219-5)
Supplement: Supplementary file 3 — Supplementary Material 3 [file 41598_2026_50219_MOESM3_ESM.docx]

**Supplemental Material**

**Design of Morphing Patterns in Knitted SMA Textile Actuators via Knitting Codes**

**Supplementary Video S1. Deformation mode of knitted textile actuators and morphing flower models**

Format: Mp4

Duration: 00:00-01:51

Resolution:1920*1080 (1080p)

Time Stamps:


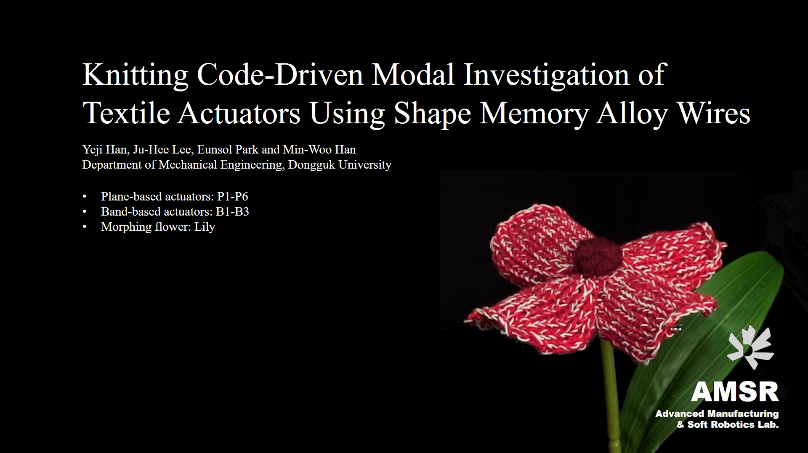


[00:00-00:04] Title frame


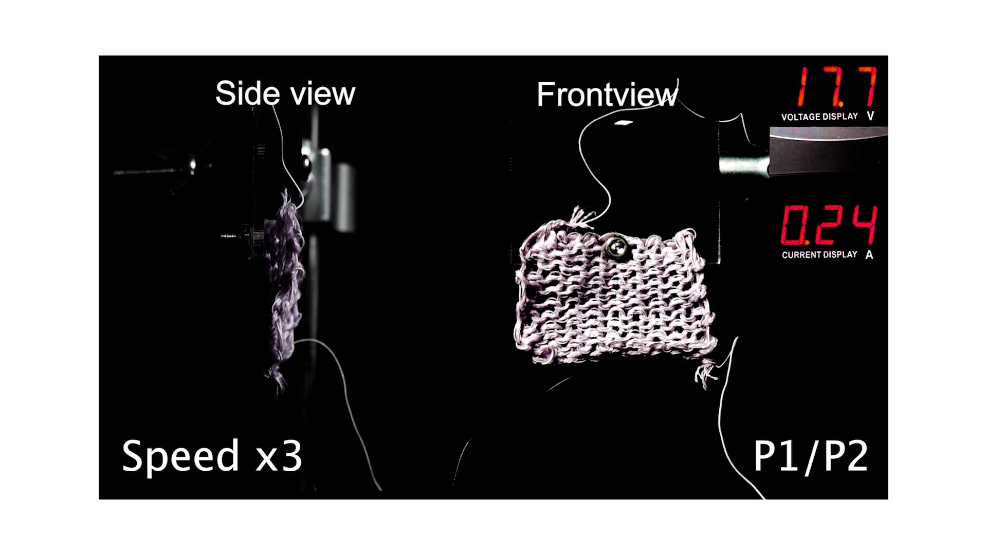


[00:05-00:53] Plane-based actuators

- Shows the deformation of plane-based actuators (P1-P6)

- Front and side views shown


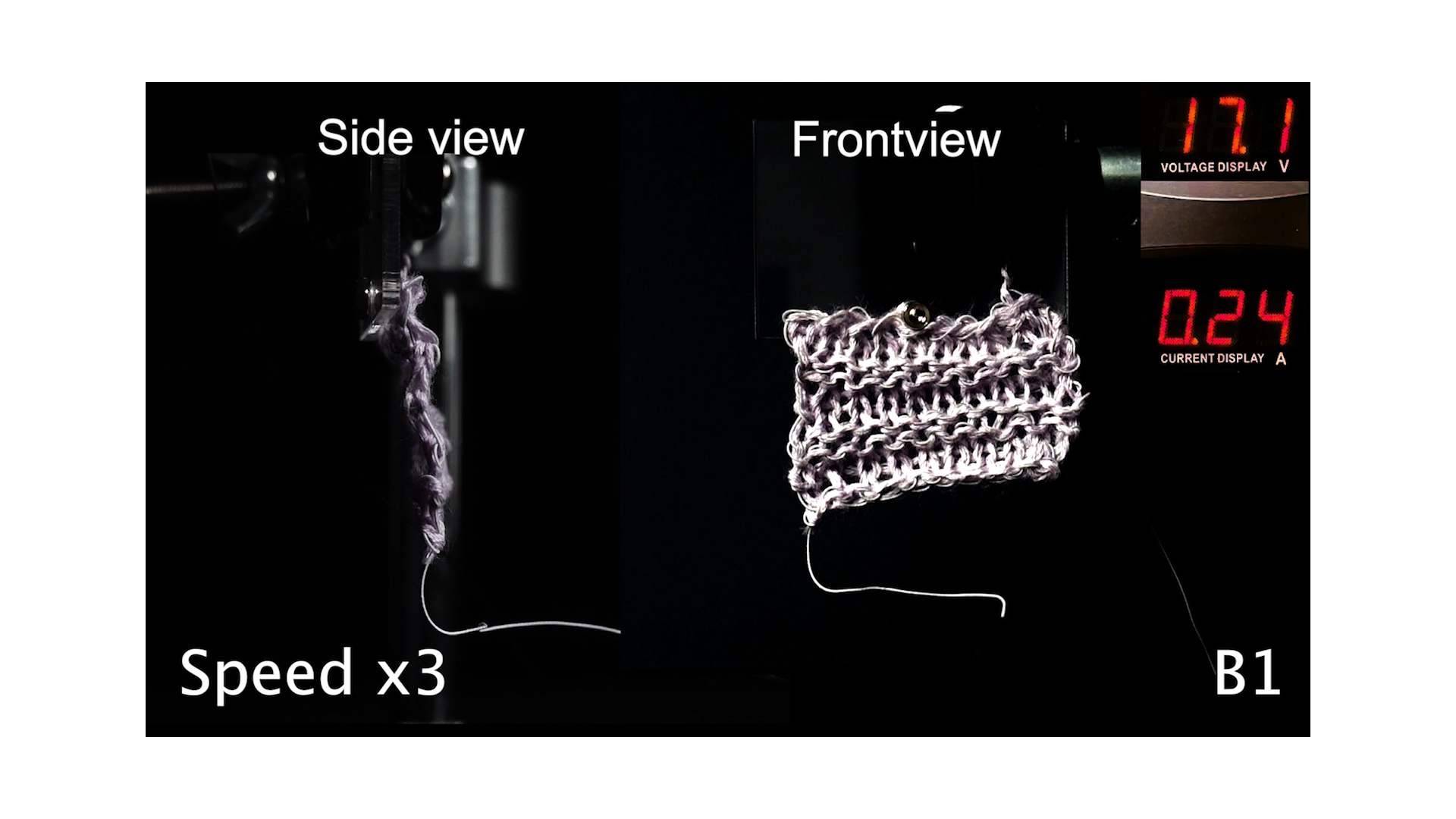


[00:58-01:39] Band-based actuators

- Shows the deformation of band-based actuators (B1-B3)

- Front and side views shown


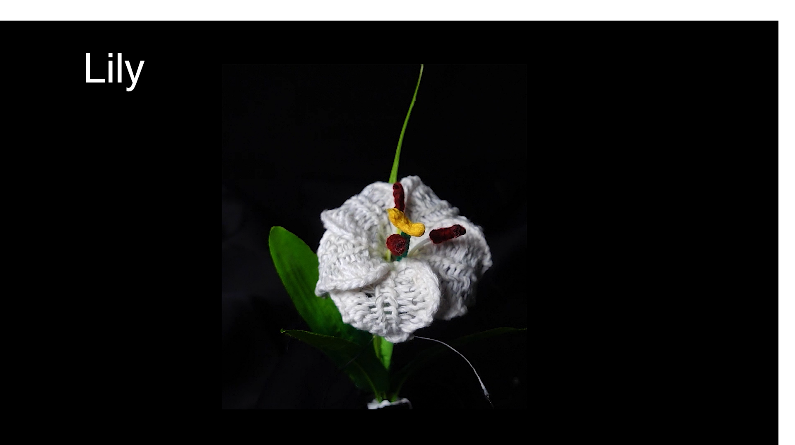


[01:40-01:51] Lily model demonstration

- Five parallel-connected petals

- Initial bending deformation prior to electrical activation

- Shows contracting deformation along course direction

- Bending deformation straightened in wale direction

**Supplementary Table S1. Technical characteristics of SMA wires with different diameters (Flexinol®, Dynalloy Inc.)** ^38^

| Diameter Size (mm) | Resistance ( **Ω**/m) | Heating Pull Force (g) | Cooling Deformation Force (g) | Approximate (mA) | Cooling Time, 70°C  Low Temperature Wire (s) |
| --- | --- | --- | --- | --- | --- |
| 0.1 | 126 | 143 | 57 | 200 | 1.1 |
| 0.2 | 29 | 570 | 228 | 660 | 3.2 |
| 0.31 | 12.2 | 1280 | 512 | 1500 | 8.1 |

**Supplementary Table S2. Electrical characteristics of basic knitted actuators based on different knitting codes under a unified actuation condition**

**-** Electrical resistance and calculated Joule heating $\left( I^{2}Rt \right)$ of knitted actuators with different knitting codes, measured under a common actuation condition of 0.25 A for 30 s.

| Knitting Code | Resistance (**Ω**) | Current (**A**) | Voltage (V) | Heating Duration (s) | Power (W) | Joule Heating  (J) |
| --- | --- | --- | --- | --- | --- | --- |
| P1/P2 | 54.38 | 0.25 | 15.9 | 30 | 3.975 | 101.9625 |
| P3 | 63.45 | 0.25 | 17.6 | 30 | 4.4 | 118.96875 |
| P4 | 56.65 | 0.25 | 15.5 | 30 | 3.875 | 106.21875 |
| P5 | 62.27 | 0.25 | 14.7 | 30 | 3.675 | 116.75625 |
| P6 | 55.47 | 0.25 | 15 | 30 | 3.75 | 104.00625 |
| B1 | 63.9 | 0.25 | 15 | 30 | 3.75 | 119.8125 |
| B2 | 54.45 | 0.25 | 15.2 | 30 | 3.8 | 102.09375 |
| B3 | 55.82 | 0.25 | 15.6 | 30 | 3.9 | 104.6625 |

**Supplementary Table S3. Electrical characteristics of morphing flower actuators (Lily and Pansy)**

**-** Electrical characteristics of a single petal of the Lily actuator at full flattening.

| Resistance (**Ω**) | Current (**A**) | Voltage (V) | Time to Full Actuation (s) | Power (W) | Joule Heating  (J) |
| --- | --- | --- | --- | --- | --- |
| 32.02 | 0.25 | 8.8 | 50 | 2.2 | 100.0625 |
| 32.02 | 0.3 | 10.3 | 21 | 3.09 | 60.5178 |
| 32.02 | 0.35 | 12 | 14 | 4.2 | 54.9143 |
| 32.02 | 0.4 | 13 | 8 | 5.2 | 40.9856 |

- Electrical characteristics of a single petal of the Pansy actuator at a curvature of 0.102 mm⁻¹.

| Resistance (**Ω**) | Current (**A**) | Voltage (V) | Time to Full Actuation (s) | Power (W) | Joule Heating  (J) |
| --- | --- | --- | --- | --- | --- |
| 64.48 | 0.25 | 18.7 | 44 | 4.675 | 177.32 |
| 64.48 | 0.3 | 22 | 22 | 6.6 | 127.6704 |
| 64.48 | 0.35 | 24.9 | 15 | 8.715 | 118.482 |
| 64.48 | 0.4 | 29 | 9 | 11.6 | 92.8512 |

**Supplementary Figure S1. Machine knitting of SMA wire using a Brother knitting machine**

**
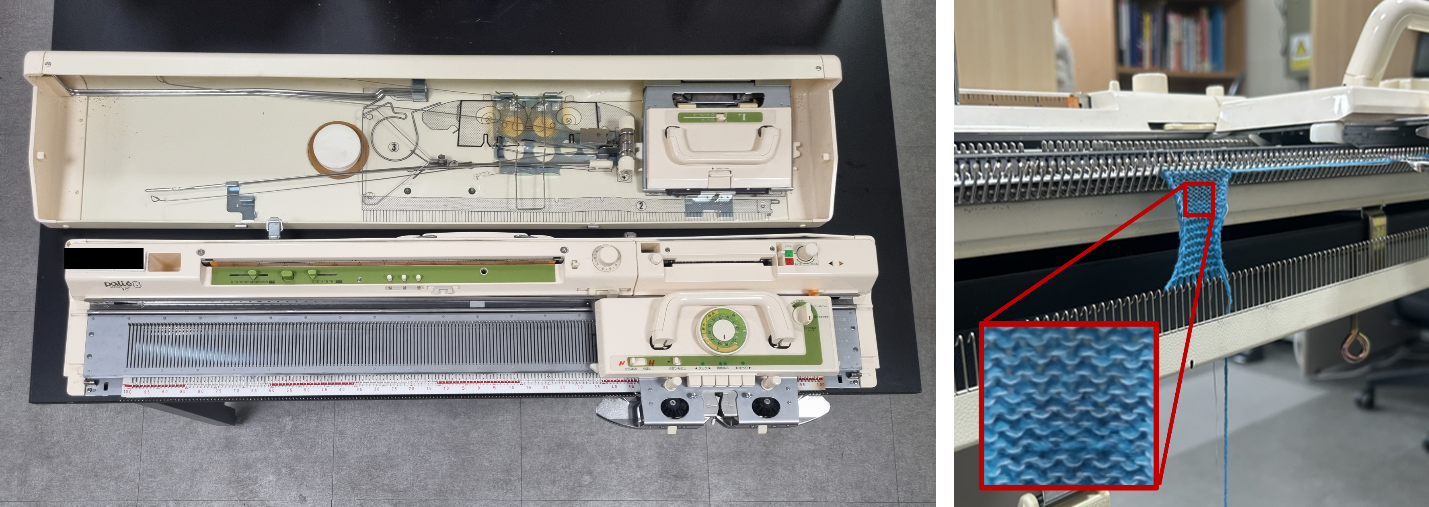
**

- Left: Photograph of the Brother knitting machine used for fabrication.

- Right: Knitted sample fabricated using the machine, consisting of wrapped SMA wire (white) and inactive fiber (blue).

**Supplementary Figure S2. Cooling rate comparison of knitted SMA wire actuators with and without inactive fiber**


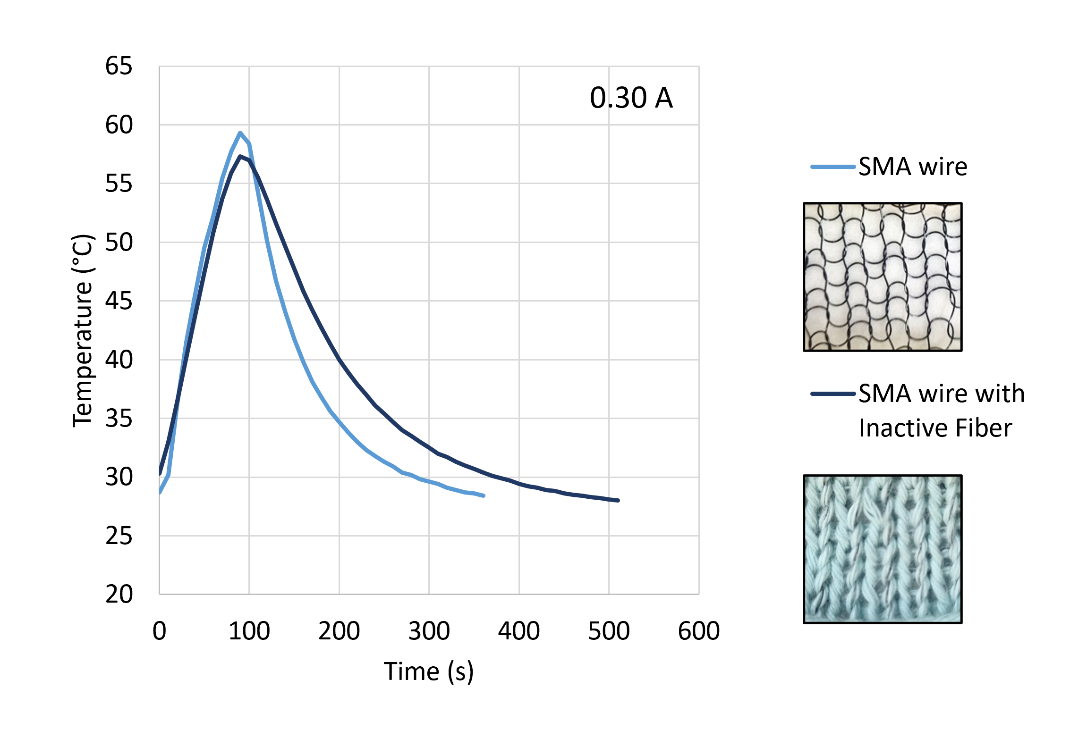


- Temperature characteristics of two 10×10 knitted actuators under 0.30 A: bare SMA wire only, and SMA wire knitted with inactive fiber. The addition of surrounding fiber reduces the cooling rate, indicating that fiber content influences thermal dissipation. (Measured using an Elitech data logger, TlogB100EC.)

- The actuators were heated for 90 s, followed by natural cooling at ambient conditions.

**Supplementary Figure S3. Temperature response of knitted actuator (P1/P2) under different Joule heating currents**


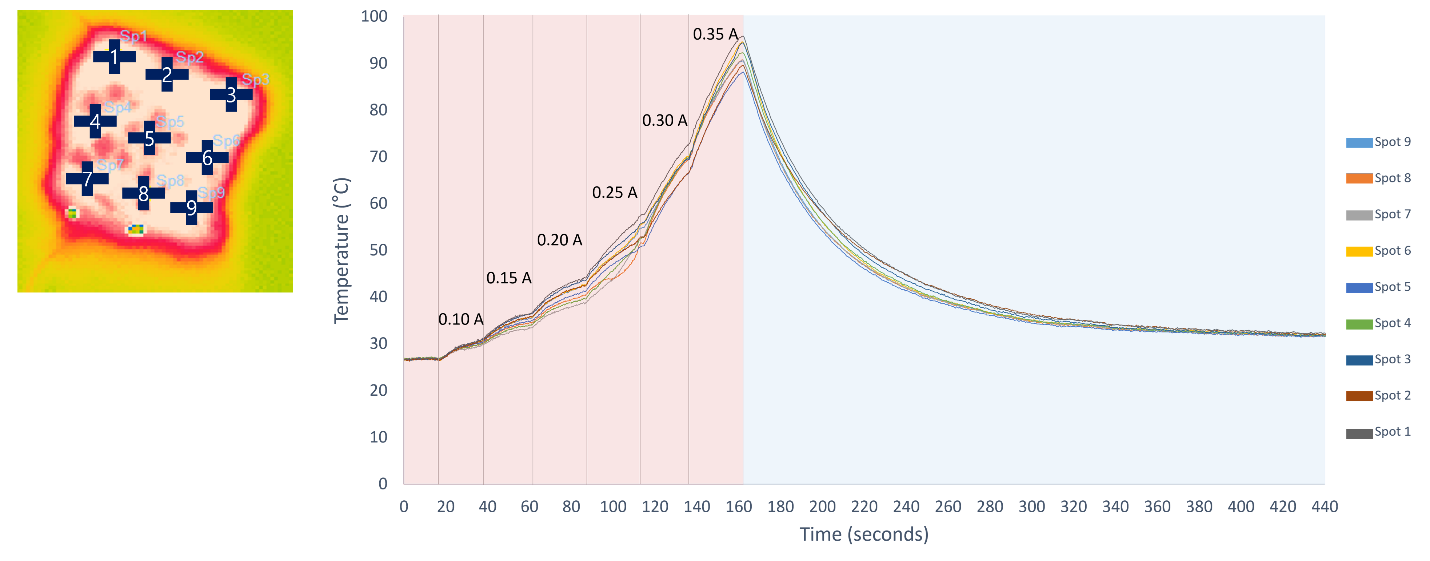


- Left: Infrared thermal image showing the locations of nine measurement spots (Sp1–Sp9).

- Right: Temperature curves of each spot under applied currents from 0.1 A to 0.35 A, followed by natural cooling.

**Supplementary Figure S4. Schematic of the parallel electrical configuration of the morphing flower actuator**

**
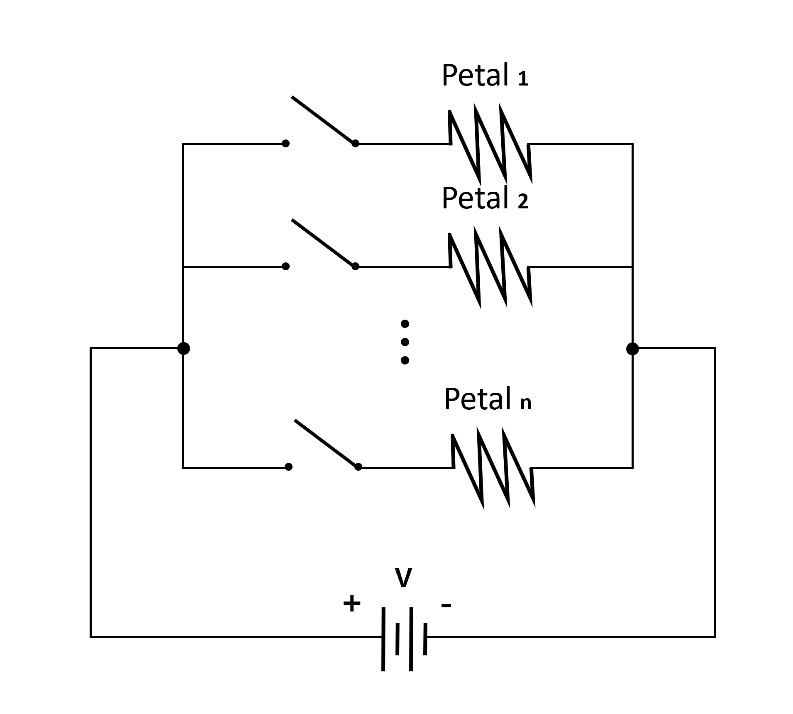
**

**Supplementary Data S1. Raw time–force data underlying Figure 6(b) and 6(e)**

Format: Excel (.xlsx)

This file contains the time–actuation force datasets measured for the P1, P3, and B1 knitted SMA textile actuators under constant current Joule heating conditions.

The data include:

- Time (s)
- Actuation force (N)
- Sample type (P1, P3, B1)
- Measurement direction (Vertical / Horizontal)

Experimental conditions:

- Applied current: 0.25 A (constant current mode)
- Ambient temperature: ~23–25 °C
- Sampling interval: 0.0246 s
- Actuator size: 10 courses × 10 wales
- SMA wire: Ni–Ti (core diameter 200 μm)

Measurement equipment:

- Load cell: ROBOTUS RFT40-SA01
- Power supply: KEITHLEY 2260B-250-4
